# KDM5C Represses FASN-Mediated Lipid Metabolism to Exert Tumor Suppressor Activity in Intrahepatic Cholangiocarcinoma

**DOI:** 10.3389/fonc.2020.01025

**Published:** 2020-06-29

**Authors:** Bo Zhang, Bing-hai Zhou, Min Xiao, Hui Li, Lei Guo, Meng-xi Wang, Shan-he Yu, Qing-hai Ye

**Affiliations:** ^1^Department of Liver Surgery and Transplantation, Liver Cancer Institute, Zhongshan Hospital, Fudan University and Key Laboratory of Carcinogenesis and Cancer Invasion (Fudan University), Ministry of Education, Shanghai, China; ^2^Shanghai Ji Ai Genetics and IVF Institute, The Obstetrics and Gynecology Hospital of Fudan University, Shanghai, China; ^3^State Key Laboratory of Medical Genomics, National Research Center for Translational Medicine at Shanghai, Shanghai Institute of Hematology, Ruijin Hospital Affiliated to Shanghai Jiao Tong University School of Medicine, Shanghai, China

**Keywords:** KDM5C, FASN, cell proliferation, cell invasion, intrahepatic cholangiocarcinoma

## Abstract

**Background:** KDM5C is a histone H3K4-specific demethylase, which has multiple biological functions during development and disease. However, the role of KDM5C in intrahepatic cholangiocarcinoma (ICC) remains unknown.

**Methods:** Expression levels of KDM5C in ICC patients were determined by qRT-PCR, western blotting and immunohistochemical assay. The functions of KDM5C in cell proliferation and invasion were determined in human ICC cells and mouse xenograft model using KDM5C overexpression and knockdown strategies *in vivo*. RNA-seq analysis was applied to investigate the transcriptional program of KDM5C. In addition, ChIP-qPCR was used to determine the regulation of FASN by KDM5C.

**Results:** Here, we show that KDM5C was downregulated in human ICC, where its diminished expression was associated with poor prognosis. ICC cell proliferation and invasion were inhibited by KDM5C overexpression. Moreover, KDM5C suppressed ICC proliferation and metastasis *in vivo*. RNA-sequencing showed that KDM5C inhibits key signal pathways of cell proliferation, cell invasion and fatty acid metabolism. ChIP-qPCR revealed that overexpression of KDM5C led to the reduction of H3K4me3 on the promoter and the corresponding downregulation of the expression of *FASN*, which represents the major target gene of KDM5C to mediate the proliferation and invasion of ICC cells.

**Conclusions:** Our results revealed the role of KDM5C as a novel tumor suppressor in ICC largely by repressing FASN-mediated lipid acid metabolism and thus KDM5C may contribute to the pathogenesis of ICC.

## Introduction

Intrahepatic cholangiocarcinoma (ICC) accounts for 10–20% of newly diagnosed liver cancers, which is the second most common primary malignancy in the liver only after hepatocellular carcinoma (HCC) (1, 2). ICC exerts destructive effects on gastrointestinal tract, and the mortality rates are almost equal to the incidence rates (1, 3). Much interest has recently been attached to epigenetic remodeling, transcriptional regulation and cell metabolism of ICC cells (4). Dysregulation of epigenetic modifications especially DNA methylation and histone modifications induces aberrant gene expression, making individuals prone to diet-related disorders, such as cancer (5). Abnormal histone modifications can be associated with metabolism-related tumors, reinforcing the concept that histone modifiers have a critical role in these processes (6–9). Thus, elucidating the pathogenesis of ICC, encompassing epigenome destabilization and metabolic disturbance, may facilitate the development of targeted therapies.

Previous research has established that dynamic histone modifications by histone lysine methyltransferases (KMTs) and demethylases (KDMs) play a key role in a variety of biological processes such as cell differentiation and tumor aggressiveness (10). Histone demethylases (KDMs) that contain the Jumonji-C (JmjC) domain catalyze demethylation of histone mainly (11). Lysine-specific histone demethylase 5C (KDM5C) (also called JARID1C) is a family member of JmJC-KDMs, which specifically catalyzes H3K4me3/me2 demethylation and inhibits gene transcription by decreasing H3K4 methylation (12). In the aspect of cancer, KDM5C has both oncogenic and anti-oncogenic properties like a double-edged sword. KDM5C overexpression predicts poor prognosis in HCC, where it has been implicated as an oncogene promoting cell invasion and metastasis (13). KDM5C has also been observed to be overexpressed and promote tumor growth in prostate cancer (14). In contrast, KDM5C plays a tumor-suppressing role in cervical cancer, breast cancer and renal carcinoma (15–17). Nevertheless, the exact effect of KDM5C on ICC is unclear.

Recent studies have found that altered lipid metabolism is a new hallmark of cancer (18–20). Enhanced lipid synthesis, uptake, and storage contribute to rapid tumor growth and malignant progression (21). Lipids act as basic structures of cancer cell membranes, besides, they can also function as signaling molecules and energy source.

Exogenous intake and *de novo* fatty acid synthesis provide two sources for fatty acids (FA), which produce many lipids. Both energy generation via fatty acid oxidation (FAO) and protein post-translational modifications of cancer cells rely on *de novo* fatty acid synthesis (22, 23). Tumor cells will depend on exogenous fatty acids, if anabolic pathways can not meet the rapid growth requirements (24). Fatty acid synthase (FASN), which catalyzes the synthesis of palmitate and 16-carbon long fatty acid from acetyl-CoA and malonyl-CoA, is a critical enzyme responsible for *de novo* fatty acid synthesis. Previous studies found that FASN is strongly upregulated in cancers such as prostate cancer, colorectal cancer, bladder cancer, ovary cancer, and lung cancer (25–30). To our knowledge, the relationship between KDM5C and FASN has not been studied.

In this study, we demonstrate that ICC patients have dramatically lower expression of KDM5C, which was consistent with the finding that KDM5C overexpression inhibits proliferation and invasion of ICC cells, which is largely ascribed to its modulation of FASN expression. These results indicate that KDM5C has an important role in the pathogenesis of ICC.

## Materials and Methods

### Patients, Clinical Samples, and Follow-Up

We collect a total of 190 ICC samples, 18 pairs of ICC tissues and its matched normal adjacent liver tissues from patients who had undergone curative hepatectomy at Zhongshan Hospital, Fudan University (Shanghai, China) from January 2006 to December 2010. The 190 samples were used for IHC assay and the 18 pairs of matched tissues were used for RT-qPCR assay, respectively. Previous studies have described the follow-up procedures (31). We obtained the written informed consent from each patient before surgery. The Zhongshan Hospital Research Ethics Committee approved the study protocol. The entire study process complied with the ethical guidelines of the 1975 Declaration of Helsinki (as revised in Brazil in 2013).

### Immunohistochemistry (IHC) and Tissue Microarray (TMA)

IHC staining procedures on tumor arrays were performed as described in previous studies (32). LeicaQWin Plus v3 software (Leica) was used to capture photographs of 3 representative fields. Image-Pro Plus v6.0 (Media Cybernetics) software was used to quantify the intensity of these proteins. We calculated the ratio of integrated optical density of positive staining to total area of each photograph as the expression intensity of these proteins as described in previous studies (33). The cut off of KDM5C or FASN was defined as the median of the values.

### Cell Culture, Plasmids, and Transfection

RBE and HCCC9810 cell lines were obtained from the Cell Bank of Shanghai Institutes of Biological Sciences, Chinese Academy of Sciences. HuH28, HuCCT1, and CCLP1 were provided by Prof. Qiang Gao (Liver Cancer Institute, Zhongshan Hospital, Fudan University). RBE and HCCC9810 were cultured in Dulbecco's modified Eagle's medium (Gibco, 31885–023) and CCLP1 were cultured in 1,640 medium (Gibco, 72400047) supplemented with 10% fetal bovine serum (Gibco, 10099–141), 20 U/mL penicillin and 20 μg/mL streptomycin (Sigma-Aldrich), with a 5% CO2 concentration at 37°C. HuH28 was cultured in MEM medium (Gibco, 42360032) supplemented with 15% fetal bovine serum (Gibco, 10099–141), 20 U/mL penicillin and 20 μg/mL streptomycin (Sigma-Aldrich), with a 5% CO_2_ concentration at 37°C.

Full-length cDNA encoding human KDM5C and FASN were cloned into pCDH-CMV-MCS-EF1-Puro (CD510B-1, System Biosciences) with or without Flag-tagged using standard protocols. The shRNA sequences for KDM5C and FASN were purchased from Sigma-Aldrich and cloned into pLKO.1 TRC (Addgene plasmid 10,879). Details about the sequences used here can be found in Table S3. We used a scrambled siRNA precursor (Scr) as control. DNA sequencing and western blot were performed to verify the constructions. Plasmids and shRNAs were transfected into cell lines through lipofectamine 2,000 (Invitrogen) like previous descriptions (32).

### Colony Formation Assays and Proliferation Assays

ICC cells were seeded at a density of 1,000 per well in six-well plates and cultured for 14 days, and then fixed and stained with crystal violet. Only colonies, clusters of more than 50 cells and visible to the naked eye, were counted.

The cell proliferation of CCLP1 and HCCC9810 cells were detected by using Cell Counting Kit-8 (Dojindo, Kumamoto, Japan) as described previously (34). Each experiment was performed for more than three times.

### Oxygen Consumption Rates Assay

A Seahorse Bioscience XFe 96 analyzer (Seahorse Bioscience, North Billerica, USA) was used to perform the OCR assays according to the manufacturer's instructions. ICC Cells were seeded into the Seahorse 96-well plate at a density of 1.5 × 10^4^ cell/well. Twelve hour later, we added 1 μM oligomycin, 1 μM FCCP and 0.5 μM Rotenonr/antimycin A into different ports of the Seahorse cartridge as described previously (35).

Each point was the average of six independent measurements.

### Chromatin Immunoprecipitation (ChIP)

We performed ChIP assays essentially as described previously (36). In brief, we harvested and crosslinked the cells with 1% formaldehyde for 10 min at room temperature. After sonication, we incubated the soluble chromatins with the following antibodies separately: anti-H3K4me3 (ab8580, Abcam); anti-KDM5C (ab34718, Abcam) or control IgG (ab172730, Abcam). Chromatin immunocomplexes were then precipitated with Protein A (16–661, Millipore) or Protein G (Millipore, 16–662). The immunoprecipitated complex was washed, and DNA was extracted and purified by QIAquick PCR Purification Kit (Qiagen). ChIP DNA was analyzed by qPCR using specific primers, and the data were normalized by input DNA. The primers used for ChIP-qPCR were as follows: human *FASN* (5′-ACAAAGGTGGAGATGGAGCT-3′, 5′-TCGGAGAACTTGCAGGAGT-3′).

### *In vivo* Tumor Growth and Metastasis Assays

The Animal Ethics Committee of Shanghai Medical College, Fudan University approved all animal experiments. We injected HCCC9810 cells overexpressed with KDM5C or empty vector as control subcutaneously into the flanks of 5 weeks old male nude mice (6 × 10^6^ cells/mouse, *n* = 8 for each group).

We measured tumor volumes every 4 days. Mice were sacrificed 28 days after injection. Xenograft tumors were collected and tumor weight was measured. To establish metastatic mouse model, we injected HCCC9810 cells overexpressed with KDM5C or empty vector as control into the intraperitoneal cavity of nude mice (3 × 10^6^ cells/mouse in 200 ul PBS).

Mice were sacrificed 6 weeks after injection. Mesenteric lymph nodes were removed and analyzed as privously (34, 37).

### Statistical Analysis

Results are expressed as mean ± SD and all statistical tests were performed as 2 sided. For data normally distributed, we performed Student's *t*-test, while the non-parametric exact Wilcoxon's signed-rank test was used to compare data not normally distributed. Cumulative survival time was estimated by the Kaplan-Meier method, and the log- rank test was applied to compare the groups. The variables in predicting the overall survival (OS) and the disease-free survival (DFS) were assessed by multivariate Cox proportional hazards regression models. *p* < 0.05 was considered statistically significant.

#### Supplemental Materials

Additional materials and methods, figures and figure legends, and supplemental tables are provided in the Supplemental Materials.

## Results

### Reduction of KDM5C Expression Is Correlated With Poor Prognosis in ICC

**Figure 1:**
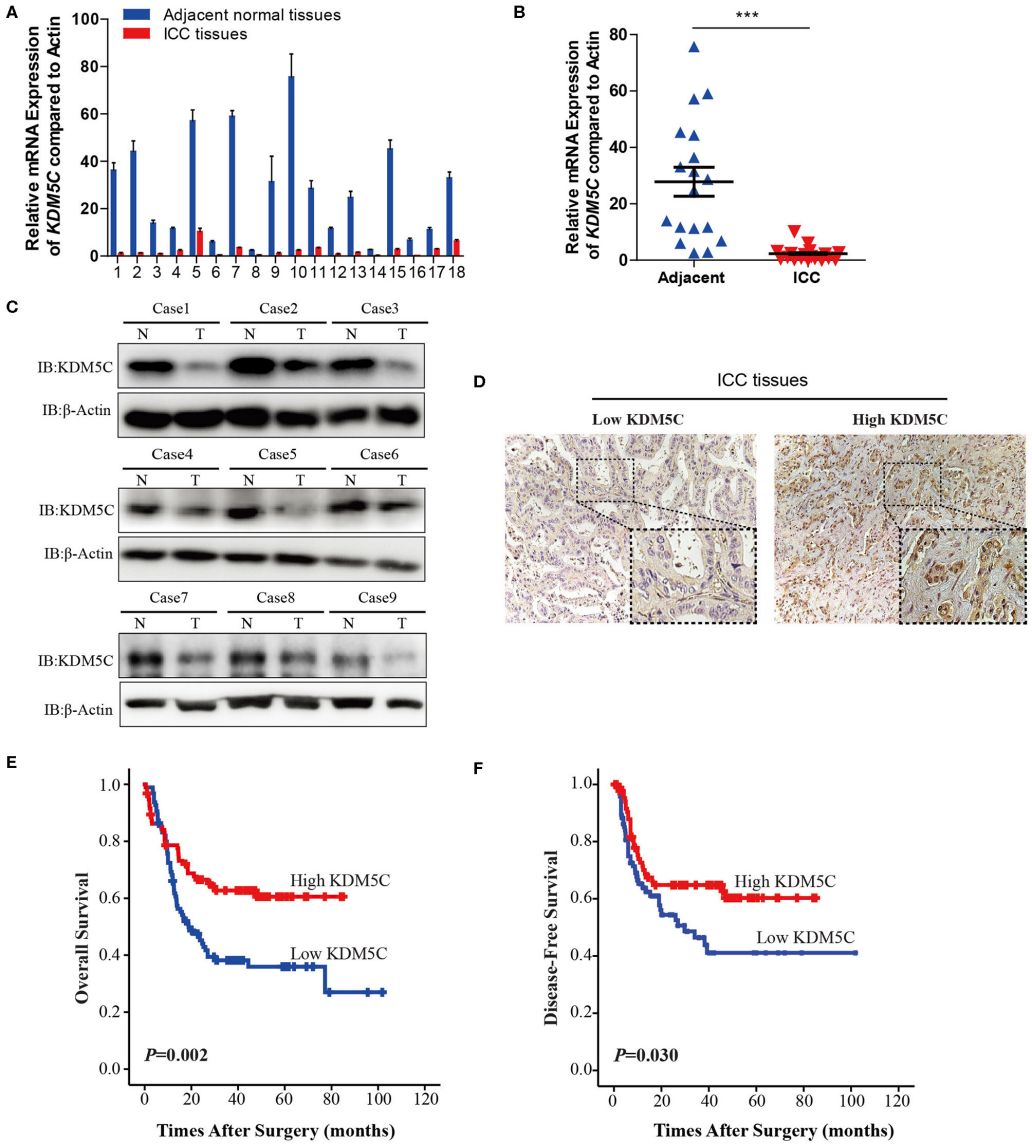
Reduction of KDM5C expression is correlated with poor prognosis in ICC. **(A)** qRT-PCR assay of *KDM5C* mRNA expression in 18 paired groups of specimens of human ICC and non-tumor adjacent tissues. **(B)** Comparison of the expression of *KDM5C* mRNA in human ICC and adjacent non-tumor liver tissues. The mRNA level of *KDM5C* was significantly downregulated in ICC tissues. **(C)** Western blot analyses of KDM5C in human ICC and adjacent non-tumor liver tissues. **(D)** Immunohistochemical patterns of KDM5C of human ICC specimens. Scale bars, 50 μm (upper) and 20 μm (lower). **(E,F)** Kaplan-Meier survival analysis of the overall survival **(E)** and disease-free survival **(F)** of ICC patients which were divided into two different groups according to the protein levels of KDM5C, which were determined by immunohistochemical assay. Data are presented as the mean ± SD. ***p < 0.001. All results are from three independent experiment.

To investigate whether KDM5C might be involved in the progression of ICC, we measured the mRNA expression level of *KDM5C* in ICC tissues (*n* = 18) and its matched normal adjacent liver tissues (*n* = 18) using qRT-PCR assays. The expression of *KDM5C* was significantly downregulated in ICC specimens, compared with normal liver tissues (Figures 1A,B). Consistently, Western blot analysis of KDM5C in ICC tissues showed the similar results (Figure 1C). We first divided the 190 cases with ICCs into two subgroups: “low KDM5C expression (*n* = 95)” and “high KDM5C expression (*n* = 95),” in order to determine the relationship between KDM5C expression and clinicopathological parameters. The representative images of high-KDM5C and low-KDM5C are showed in Figure 1D. We only found significant correlations between KDM5C expression and regional lymph node metastasis (*p* = 0.009), and tumor-node-metastasis stage of ICC (*p* = 0.013) (Table S1). Patients with high KDM5C expression had both higher overall survival (*p* = 0.002) and better disease-free survival (*p* = 0.030) (Figures 1E,F and Table S1) than the low KDM5C expression group. To sum up, these results suggest that, contrary to the increased expression of KDM5C in hepatic carcinoma (which plays a carcinogenic role), the decreased expression of KDM5C in ICC is correlated with a poor prognosis.

### Overexpression of KDM5C Inhibits ICC Growth and Metastasis *in vitro* and *in vivo*

**Figure 2:**
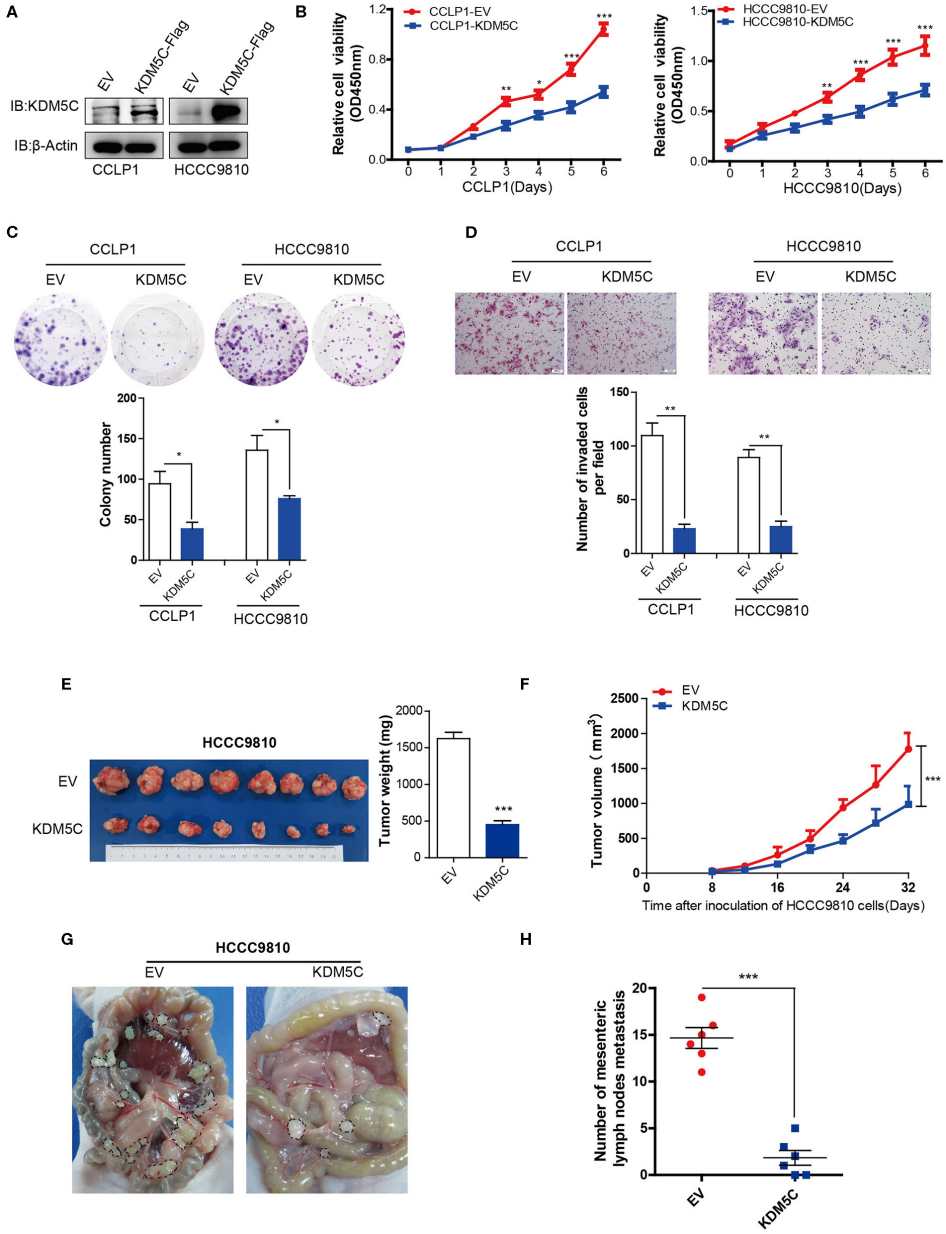
Overexpression of KDM5C inhibits ICC growth and metastasis *in vitro* and *in vivo*. **(A)** Western blotting assay of KDM5C protein in CCLP1 and HCCC9810 cells stably transduced with vector control or KDM5C-expressing vector. **(B)** Cell Counting Kit 8 assay of cell proliferation after KDM5C overexpression in CCLP1 and HCCC9810 cells. **(C)** Colony formation assay and statistical analysis of the colonies of CCLP1 and HCCC9810 cells upon overexpression of KDM5C. **(D)** Matrigel invasion assay and statistical analysis of invaded cells of CCLP1 and HCCC9810 cells upon overexpression of KDM5C. Scale bars, 100 μm. **(E)** HCCC9810 cells transduced with vector control or KDM5C-expressing vector and subcutaneously injected into nude mice, mice were sacrificed after 32 days and the tumor weight was measured. **(F)** The dynamic changes of tumor volume in subcutaneous models was shown at 32 days after injection. **(G,H)** Representative intraperitoneal metastasis and the number of mesenteric lymph nodes metastases after KDM5C overexpression in HCCC9810 cells. Data are presented as the mean ± SD. *p < 0.05, **p < 0.01, ***p < 0.001. All results are from three independent experiments.

The functional relevance of KDM5C to the biological behaviors of ICC was then investigated *in vitro* and *in vivo*. First, we analyzed the expression level of KDM5C in a panel of ICC cell lines. KDM5C protein levels of HuCCT1 and CCLP1 cells with high potentiality of metastasis were much lower than that of the other with low potentiality of metastasis (Figure S1). To investigate the role of KDM5C in ICC, CCLP1 and HCCC9810 cells were lentivirally transduced with empty control or KDM5C-expression cassette to achieve stable over-expression, respectively (Figure 2A). Cell proliferation assays showed that overexpression of KDM5C strongly decreased cell proliferation in CCLP1 and HCCC9810 cells (Figure 2B). Consistently, KDM5C overexpression decreased the colony-forming potential of these two ICC cell lines. (Figure 2C) Furthermore, Matrigel invasion assays revealed that KDM5C overexpression significantly weakened the invasion ability of ICC cells (Figure 2D).

To further explore the role of KDM5C *in vivo*, we injected HCCC9810 cells overexpressed with empty control or KDM5C into immunocompromised nude mice. Critically, the tumor sizes of the xenografts were significantly smaller in KDM5C-overexpressed group than control (Figures 2E,F). We injected KDM5C-overexpressed HCCC9810 cells into the intraperitoneal cavities of nude mice, to further evaluate the effects of KDM5C on metastasis. We counted the number of metastatic mesenteric lymph nodes per mouse and discovered that overexpression of KDM5C could significantly reduce the metastases to mesenteric lymph nodes (Figures 2G,H). Thus, these results suggested that KDM5C inhibits tumor growth and metastasis of ICC.

### KDM5C Inhibits Key Regulators of Cell Proliferation, Cell Invasion, and Fatty Acid Metabolism

**Figure 3:**
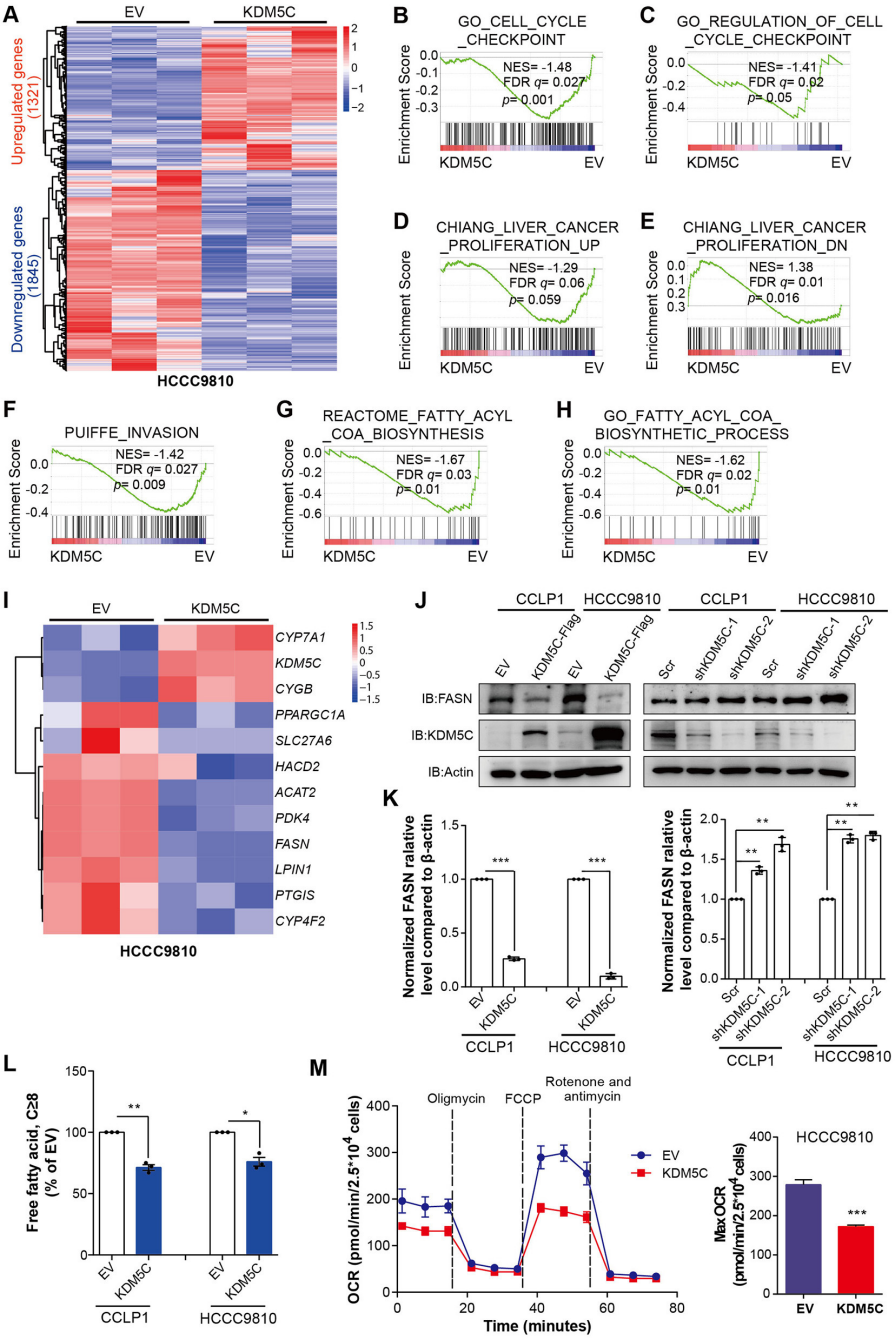
KDM5C inhibits key regulators of cell proliferation, cell invasion and fatty acid metabolism. **(A)** Heatmap showing the differentially expressed genes between HCCC9810 cells stably transduced with control or KDM5C-expressing vector (fold change ≥1.5, *p* < 0.05). **(B–H)** GSEA of the expression profile of HCCC9810 cells upon overexpression of KDM5C using cell cycle checkpoint-associated signatures **(B,C)**, liver cancer proliferation-associated upregulated or downregulated signatures **(D,E)**, an invasion-associated signature **(F)** and fatty acetyl-CoA biosynthesis-associated signatures **(G,H)**. **(I)** Heatmap showing the differentially expressed genes of HCCC9810 cells upon overexpression of KDM5C, which focused on a set of genes of fatty acid metabolism. **(J,K)** FASN expression after KDM5C overexpression (in CCLP1 and HCCC9810 cells) and KDM5C knockdown (in CCLP1 and HCCC9810 cells) **(J)**, relative FASN protein levels were normalized against β-actin using paired *t*-test and EV acted as control **(K)**. **(L)** Cellular free fatty acid was measured in HCCC9810 and CCLP1 cells with EV or KDM5C overexpressed. **(M)** Oxygen consumption rates (OCR) were measured after KDM5C overexpression in HCCC9810 cells. Data are presented as the mean ± SD. *p < 0.05, **p < 0.01, ***p < 0.001. All results are from three independent experiments.

In order to better understand the mechanisms of KDM5C engaged in ICC, we performed gene expression profiling of HCCC9810 cells overexpressed with empty control or KDM5C. Our analysis identified ~3,000 differentially expressed genes after KDM5C overexpression (fold change ≥1.5, *p* < 0.05, Figure 3A and Supplementary Data 1). We performed gene set enrichment analysis (GSEA) to identify potential signaling pathways interfered by the gain of function of KDM5C, so as to gain further insights into the molecular pathways regulated by KDM5C. Consistent with the observation above, GSEA revealed significant negative enrichment for the gene signatures associated with cell cycle (Figures 3B,C), cell proliferation (Figure 3D), and cell invasion (Figures 3D–F) in KDM5C-overexpressed HCCC9810 cells. Interestingly, GSEA also showed a negative enrichment for the gene signature associated with fatty acid metabolic pathway (Figures 3G,H, Figures S2A,B). Likewise, gene ontology (GO) analyses revealed that the genes differentially expressed upon KDM5C overexpression were enriched in functional categories linked to fatty acid metabolic process (Figure S2C). Consistently, KDM5C overexpression in HCCC9810 cells altered the mRNA levels of a number of key regulators of fatty acid metabolism (Figure 3I). Genes involved in positive regulation of fatty acid metabolism including *LPIN1* (38) and *FASN* (39) were decreased by KDM5C overexpression. In contrast, the fatty acid metabolism blockers, *CYGB* and *CYP7A*1, were enhanced by KDM5C overexpression. Reprogramming of metabolic pathways in cancer cells has been shown to support cancer cell proliferation and survival (40–42). Among these downregulated fatty acid metabolic genes, the adjusted *p*-value of *FASN* ranked at the top (Figure 3I).

FASN plays an important role in palmitate synthesis which is a precursor of fatty acids, and is upregulated frequently in many human cancers (39, 43, 44). It has been proven to be essential for cancer cell survival, and its overexpression has been correlated with a poor prognosis and a higher risk of recurrence in different tumors (43–45). These data made us assume that KDM5C may work through FASN. To verify this hypothesis, we first determined whether FASN is a downstream target of KDM5C in ICC cells. The expression of FASN in ICC cells with altered KDM5C levels was further assessed by Western blot. KDM5C-overexpressing CCLP1 and HCCC9810 cells exhibited greatly decreased protein expression of FASN (Figures 3J,K), whereas silencing KDM5C dramatically increased its protein levels (Figures 3J,K). Consistent with the results obtained in KDM5C-overexpressed ICC cells, KDM5C knockdown significantly enhanced cell proliferation and invasion in CCLP1 and HCCC9810 cells (Figures S2D–G). As fatty acids directly linked to FASN function, we measured the free fatty acids in ICC cells caused by KDM5C overexpression or KDM5C knockdown. The free fatty acids were significantly downregulated by KDM5C overexpression and were upregulated by KDM5C knockdown (Figure 3L, Figure S2H). Furthermore, we monitored cellular oxygen consumption rates (OCR) by using oligomycin, FCCP, rotenone/antimycin A. We found that the OCR was significantly decreased in the KDM5C-overexpressing ICC cells compared with control cells (Figure 3M), suggesting that cellular bioenergetics metabolism was inhibited by abnormal expression of KDM5C. Therefore, we speculated that the suppression of tumor growth and metastasis of KDM5C may be associated with fatty acid metabolic pathway.

### FASN Represents the Major Target Gene of KDM5C to Regulate Cell Proliferation and Invasion

**Figure 4:**
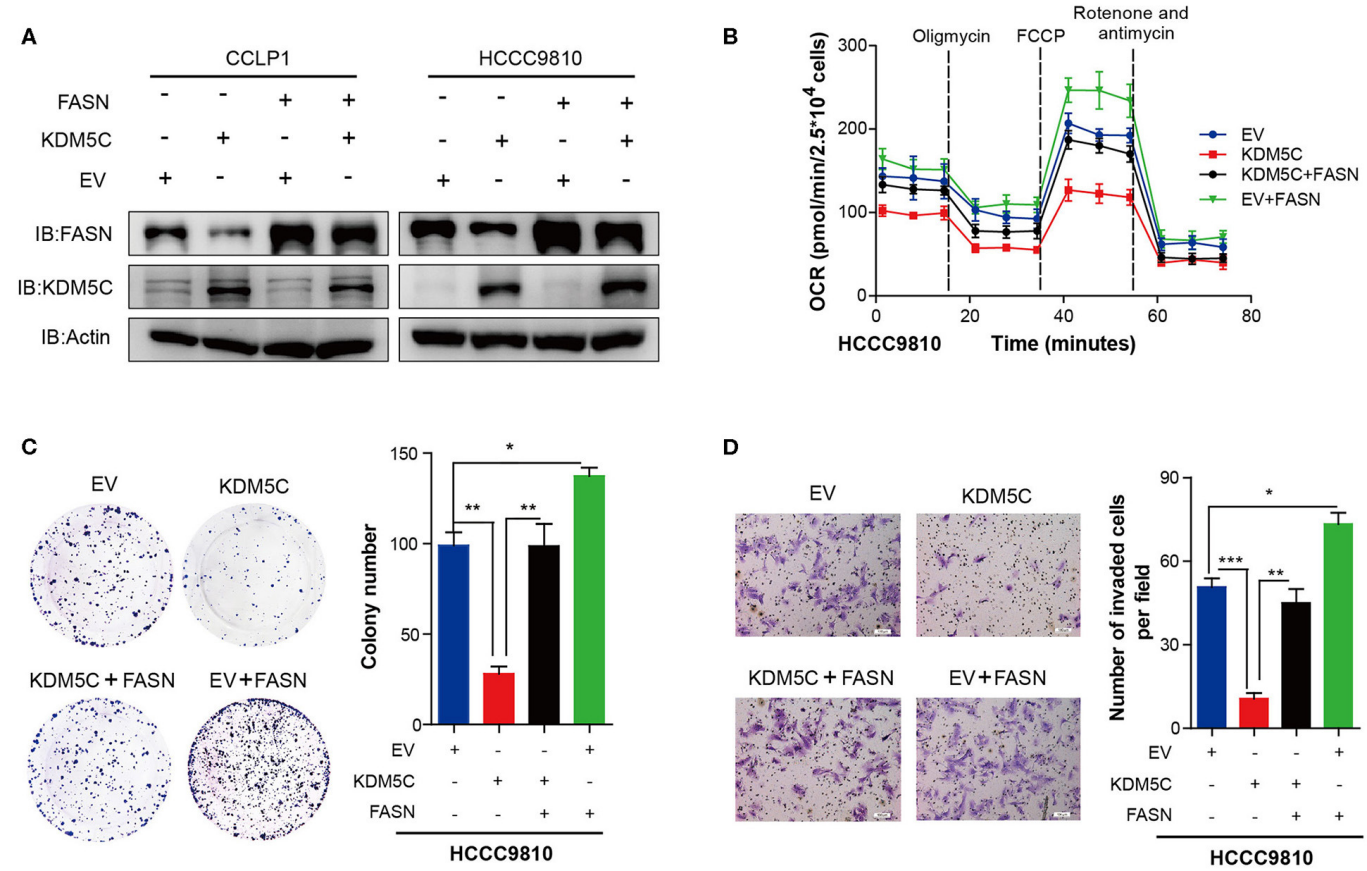
FASN represents the major target gene of KDM5C to regulate cell proliferation and invasion. **(A)** Western blotting assay of FASN and KDM5C protein of FASN and/or KDM5C overexpression experiments in CCLP1 and HCCC9810 cells. **(B)** Oxygen consumption rates (OCR) were measured after restoration of the expression of FASN in control or KDM5C-overexpressed cells. **(C)** Colony formation and mean number of colonies after restoration of the expression of FASN in control or KDM5C-overexpressed cells. **(D)** Quantification of invaded cells after restoration of the expression of FASN in control or KDM5C-overexpressed cells were shown. Scale bars, 100 μm. Data are presented as the mean ± SD. *p < 0.05, **p < 0.01, ***p < 0.001. All results are from three independent experiments.

To investigate whether KDM5C inhibits cell migration and invasion through regulating FASN expression, we restored FASN expression in KDM5C-overexpressed CCLP1 and HCCC9810 cell lines (Figure 4A). Firstly, we found the OCR was significantly increased upon restoration expression of FASN in KDM5C-overexpressed cells (Figure 4B, Figure S3A). Secondly, colony formation assay showed that overexpression of FASN could dramatically rescue the cell proliferation arrest induced by overexpression of KDM5C in HCCC9810 cells (Figure 4C). What is more, cell invasion assay revealed that overexpressing FASN significantly reversed the reduction of cell invasion ability caused by KDM5C overexpression (Figure 4D). To further conform whether the downstream metabolites of FASN could restore cell proliferation and invasion by KDM5C overexpression, we added exogenous fatty acids into the culture system of KDM5C-overexpressed ICC cells. Interestingly, the cell proliferation and invasion restrained by KDM5C overexpression could significantly rescued by adding exogenous fatty acids (Figures S3B–E). In general, these data demonstrated that KDM5C represses the proliferation and invasion of ICC cells via mediating FASN expression.

### KDM5C Regulates Dynamic H3K4me3 Modifications in the Promoter Region of FASN

**Figure 5:**
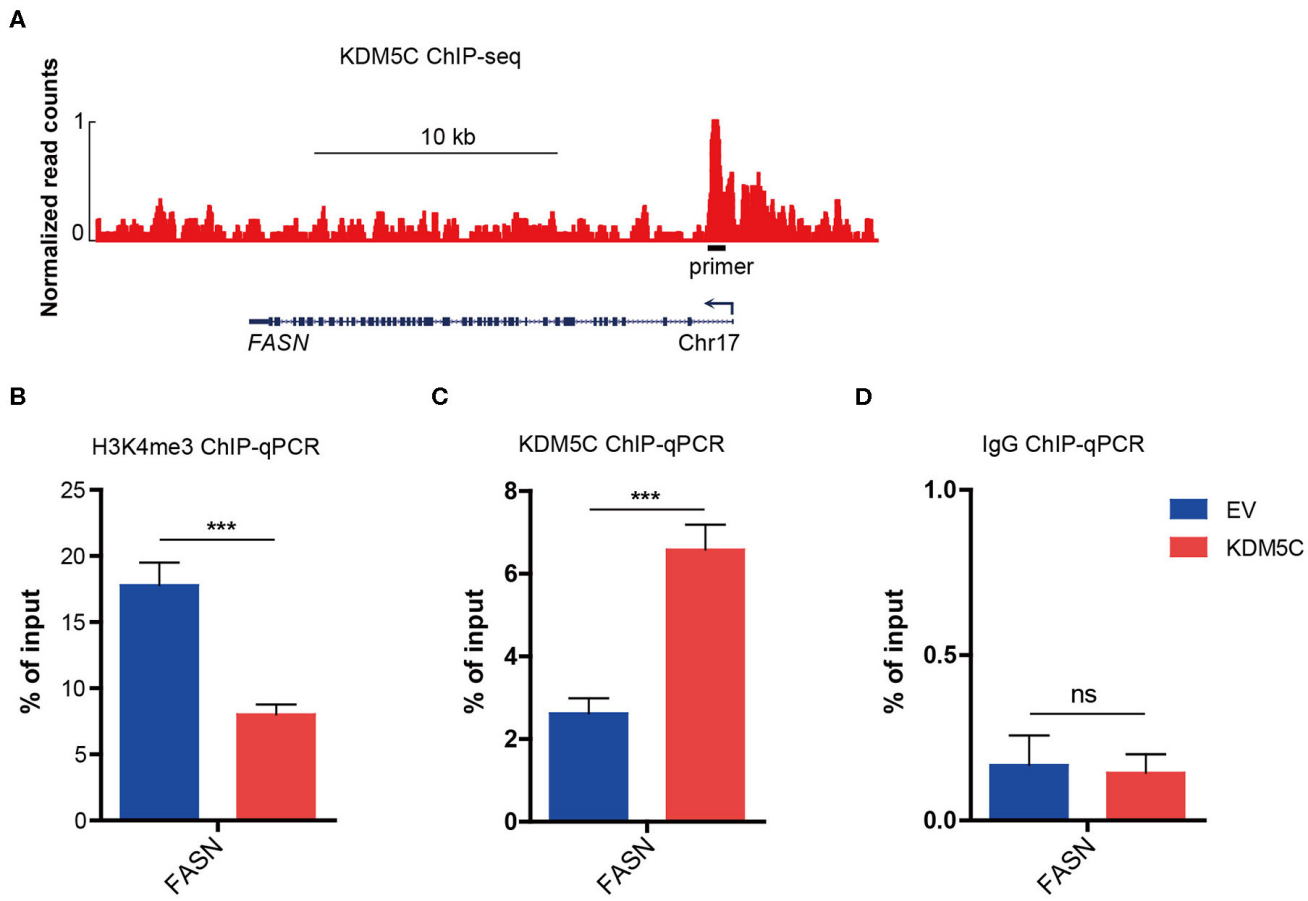
KDM5C regulates dynamic H3K4me3 modifications in the promoter region of FASN. **(A)** Genome browser track representing the binding sites of KDM5C at *FASN* gene locus in breast cancer cells. **(B–D)** ChIP-qPCR assay of H3K4me3 **(B)**, KDM5C **(C)** or IgG **(D)** occupancy at FASN gene locus in HCCC9810 cells transduced with control or KDM5C-expressing vector. Data are presented as the mean ± SD. ***p < 0.001. All results are from three independent experiments.

In order to make clear the way KDM5C regulates FASN expression, we analyzed the public ChIP-seq database which is processed by Cistrome analysis pipeline, and found a strong binding site of KDM5C in the promoter region of the gene locus of *FASN* in breast cancer cells (Figure 5A). We decided to examine the function of this region in the regulation extracted from the empty control and KDM5C-overexpressed HCCC9810 cells. ChIP-qPCR assay showed that the reduction of H3K4me3 was evident around the promoter of FASN upon KDM5C overexpression (Figure 5B). In parallel, ChIP–qPCR assay on the potential KDM5C-binding site in the promoter of *FASN*, where the H3K4me3 abundance was significantly reduced, showed that the occupancy of KDM5C was significantly accumulated by KDM5C overexpression in HCCC9810 cells (Figures 5C,D). Consistent with the predicted role of KDM5C as a transcriptional repressor through removing the histone activation marker H3K4me3, our results revealed that *FASN* is targeted directly by KDM5C during the progression of ICC.

### FASN Expression Negatively Correlates With KDM5C Expression in ICC Patients

**Figure 6:**
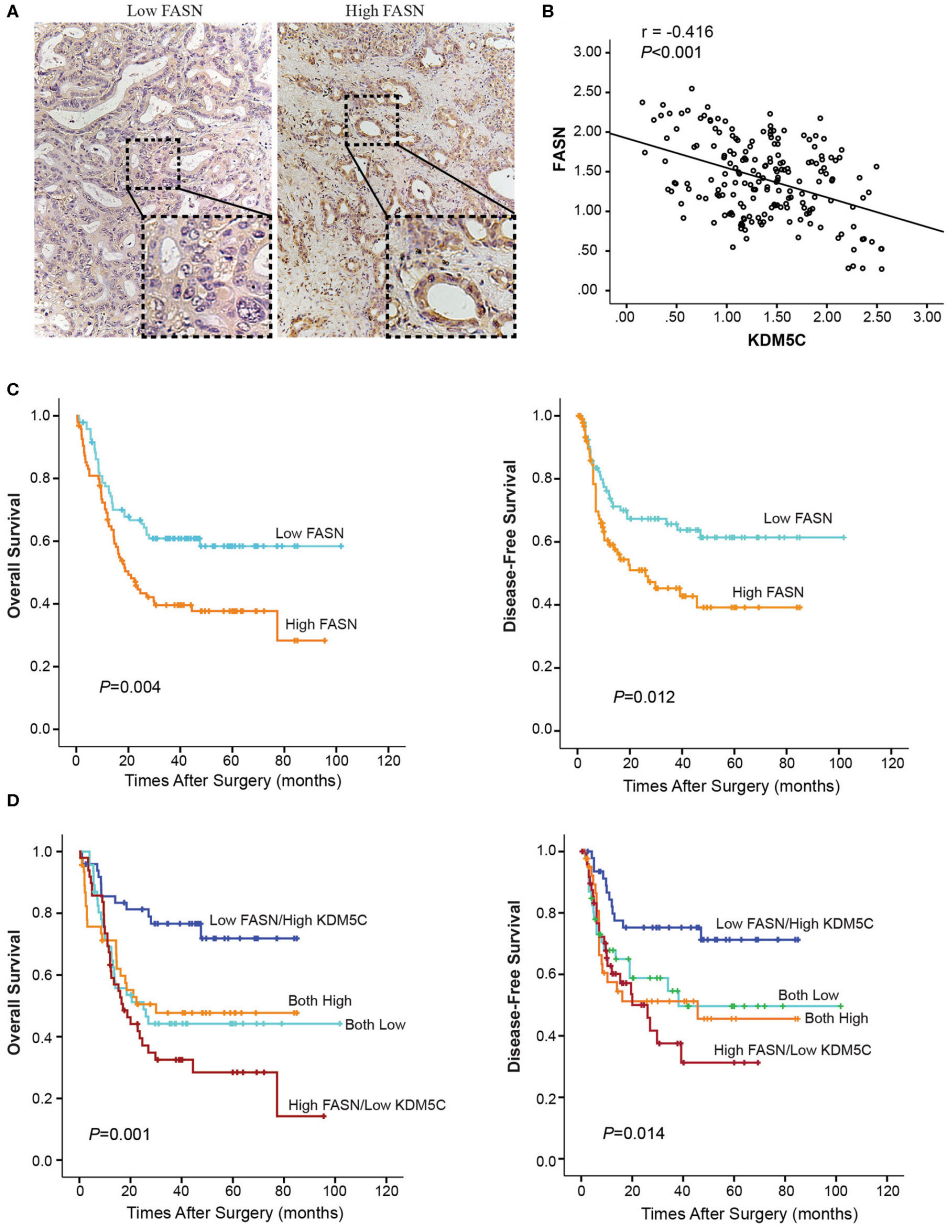
Prognostic value of KDM5C and FASN for ICC patients. **(A)** Representative immunohistochemical images of high-FASN and low-FASN expression are shown. **(B)** Spearman's correlation analysis showed that KDM5C expression level was negatively correlated with FASN expression level in ICC tissues (*p* < 0.001, *r* = −0.416). **(C)** The overall survival and disease-free survival of FASN-low and FASN-high patients were analyzed with Kaplan-Meier survival analysis. **(D)** The overall survival and disease-free survival of different groups according to the expression patterns of KDM5C and FASN were analyzed with Kaplan-Meier survival analysis. All results are from three independent experiments.

The clinical significance of KDM5C and FASN in human ICC was further investigated. Firstly, we divided the 190 cases with ICCs into two subgroups: “low FASN expression (*n* = 95)” and “high FASN expression (*n* = 95)” so as to analyze the relationship between FASN expression and clinicopathological parameters (Table S1). FASN expression and the clinicopathological parameters listed in the Table S1 had no statistical connections. The correlation between FASN expression and the OS and DFS of selected patients was analyzed with K-M survival analysis (Figures 6A,B). We found that both OS (*p* = 0.004) and DFS (*p* = 0.012) were shorter in high FASN expression group significantly than its low counterpart. Then, patients were divided into four groups according to KDM5C and FASN expression. Survival analysis showed that patients with low FASN and high KDM5C expression pattern had the longest OS (Figure 6C) and DFS (Figure 6D), whereas patients with the high FASN and low KDM5C expression pattern had the shortest OS (Figure 6C) and DFS (Figure 6D) and vice versa (*p* < 0.001, *r* = −0.416, Figure 6B). Furthermore, univariate and multivariate analyses proved FASN and KDM5C to be independent prognostic indicators for both OS (*p* = 0.003) and DFS (*p* = 0.012) (Table S2). In conclusion, these results manifested that there is a negative correlation between the expression of KDM5C and FASN. Besides, KDM5C plays a protective role in patients with ICC and can be used as a good indicator for prognosis.

## Discussion

KDM5C was reported to display a dual role as both an oncogene and a tumor suppressor (13–15, 17, 46). As an oncogene, KDM5C is found to be upregulated and promotes cell proliferation and metastasis in HCC and prostate cancer (13, 14, 46). In contrary, KDM5C acts as a tumor suppressor in clear cell renal carcinoma (ccRCC) (47), cervical cancer and breast cancers (15, 17). However, the function of KDM5C in ICC remains unknown. In our research, we discovered that the mRNA and protein expression levels of KDM5C were higher in ICC specimens than the non-tumor adjacent tissues significantly, and the downregulation of KDM5C was associated with poor prognosis in ICC, which suggested that KDM5C is somehow disrupted under pathological conditions. Furthermore, we also provided evidence to demonstrate KDM5C can inhibit the proliferation and invasion of ICC cells utilizing *in vitro* cell proliferation and invasion assay and *in vivo* experiments of ICC xenografts in nude mice. In conclusion, our results suggested that KDM5C efficiently represses cell proliferation and invasion and exerts tumor-suppressing activity in ICC cells. This is in contrast to the previous study which reported KDM5C functions as an oncogene in HCC (13). HCC and ICC are the two most common types of primary liver cancers. However, unlike HCC, ICC is a primary epithelial cancer arising within liver, which is rare, highly aggressive and often fatal (48). These observations indicated that KDM5C may have uncharacterized effects on cell survival, differentiation, proliferation and invasion, and KDM5C promotes the pathological process in a cancer cell type-dependent manner.

In cervical cancer, KDM5C represses the expression of the *EGFR* to function as a tumor suppressor (49). In this study, we observed that the *EGFR* was not significantly changed in gene expression profiling of KDM5C-overexpressed cells (Supplementary Data 1), while the fatty acid metabolic pathway were significantly restrained, suggesting that cellular bioenergetics metabolism was suppressed by the abnormal expression of KDM5C (Figures 3K,L). FASN was downregulated most significantly among all those downregulated genes (Figure 3I), which is reported to be crucial for the survival of cancer cells. Then, we found that, upon the restoration of FASN expression in KDM5C-overexpressing cells, the OCR was significantly increased, consistent with the enhanced cell growth and invasion ability. To our knowledge, this is the first research to show that KDM5C affects the biological behaviors of tumor cells by regulating fatty acid metabolism.

A hallmark of cancer is dysregulation of *de novo* lipid synthesis (50). Previous studies indicated that enhanced lipid synthesis provides energy for cancer cells and allows them to survive for a longer time (50–54). FASN catalyzes the synthesis of palmitate, a precursor of fatty acids. In different tumors, overexpression of FASN has been correlated with poor prognosis and higher risk of recurrence (50, 54). Regulation of FASN in cancers is partly owing to the transcriptional activation by SREBPs (55, 56). Additionally, FASN protein stabilization was reported to be enhanced by USP2a (50). Our results proved that downregulation of KDM5C upregulates the expression of *FASN* at mRNA level. In terms of mechanism, reduction of KDM5C increases the H3K4me3 modification in the gene promoter of FASN and thus induces its transcriptional activation. This is consistent with the function of KDM5C as specifically removes methyl residues from tri- and di-methylated lysine 4 on histone H3 lysine 4 (H3K4), and inhibits the expression of oncogenes in tumors (57). This novel regulatory effect of KDM5C on the transcriptional modification of FASN, which may be underlying the pathogenesis of ICC, can be a therapeutic target of ICC in future.

## Conclusions

In this research, we verified that KDM5C has a novel tumor-suppressing role in ICC, which inhibits the proliferation of invasion of ICC cells both *in vitro* and *in vivo*. In terms of mechanism, we proved our viewpoint by inhibiting transcriptional activation of FASN through decreasing H3K4me3 modification at FASN gene promoter and thus suppresses FASN-mediated lipid acid metabolism and the proliferation and invasion of cancer cells. Therefore, KDM5C may be involved in the pathogenesis of ICC by targeting FASN, and can be a potentially effective therapeutic target for ICC in future.

## Data Availability Statement

The datasets generated for this study can be found in the GEO repository, GSE143781. All other relevant data are available from the corresponding author on request.

## Ethics Statement

The studies involving human participants were reviewed and approved by Zhongshan Hospital Research Ethics Committee. The patients/participants provided their written informed consent to participate in this study. The animal study was reviewed and approved by Animal Ethics Committee of Shanghai Medical College, Fudan University.

## Author Contributions

BZha, BZho, and MX designed the entire study and performed the experiment. HL and LG collected patient sample‘s clinical information and analyzed data. BZha and BZho wrote the manuscript. SY and QY designed and supervised the entire project and wrote the manuscript. MW critically read the manuscript. All authors contributed to the article and approved the submitted version.

## Conflict of Interest

The authors declare that the research was conducted in the absence of any commercial or financial relationships that could be construed as a potential conflict of interest.

## Abbreviations

KDM5C: Lysine-specific demethylase 5C
ICC: intrahepatic cholangiocarcinoma
qRT-PCR: quantitative real time PCR
ChIP-qPCR: chromatin immunoprecipitation coupled with quantitative PCR
FASN: fatty acid synthase
IHC: immunohistochemistry
OS: overall survival
DFS: disease-free survival.
