# Supplementary material for: KDM5C Represses FASN-Mediated Lipid Metabolism to Exert Tumor Suppressor Activity in Intrahepatic Cholangiocarcinoma
Source: Front Oncol. 2020 Jun 29;10:1025. doi: 10.3389/fonc.2020.01025 (PMC7344276; doi:10.3389/fonc.2020.01025)
Supplement: Supplementary file 1 [file Data_Sheet_1.DOCX]

# Supplementary Figures and Tables

**Supplementary Figures**

**Figure S1**

**
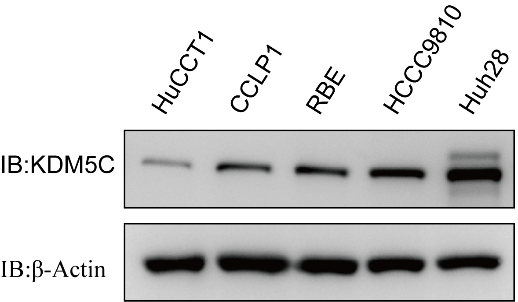
**

**Fig. S1** ICC cell lines were subjected to western blot to analyze the expression level of KDM5C using KDM5C and β-Actin antibody.

**Figure S2**

**
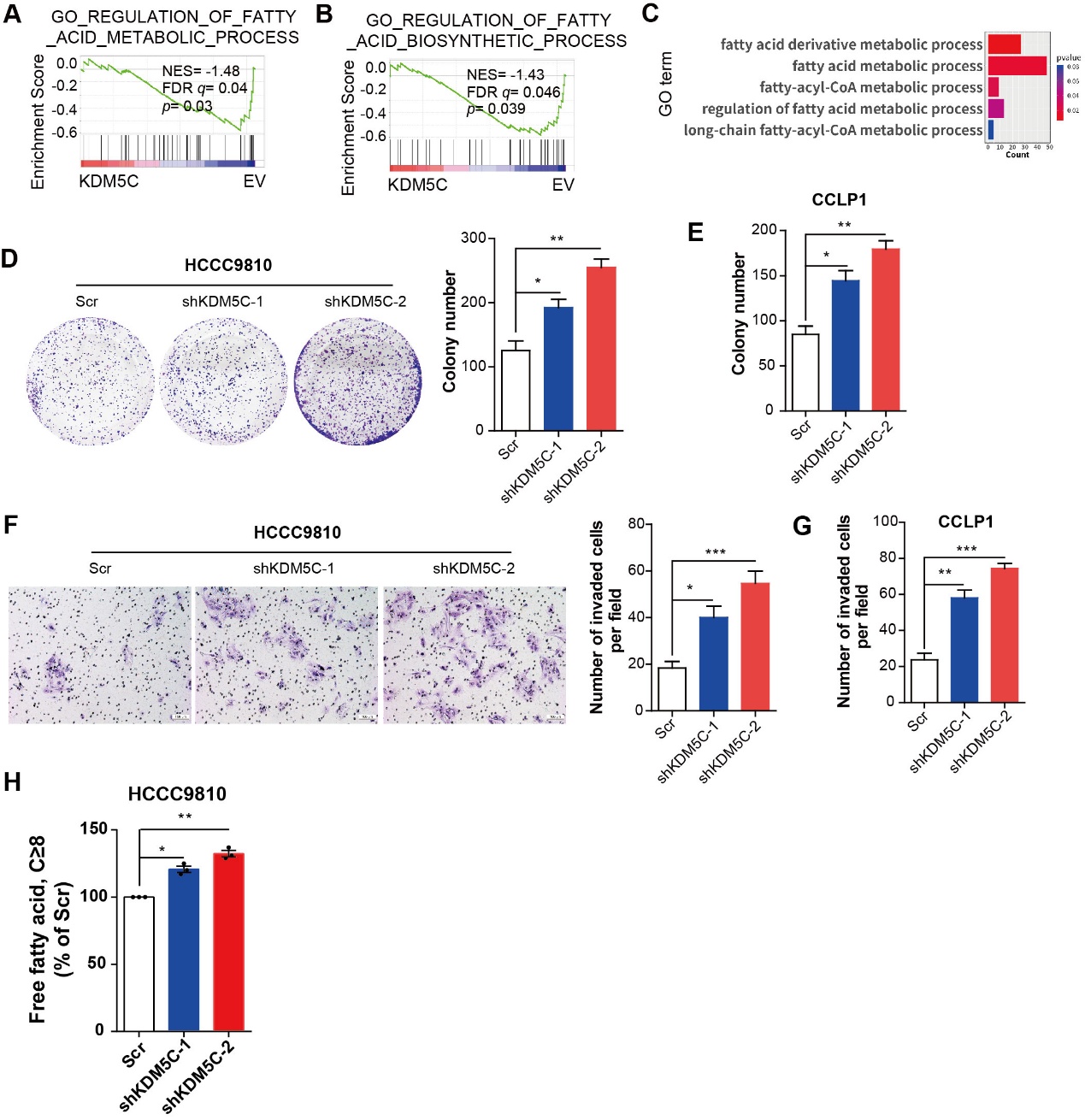
**

**Fig. S2** KDM5C inhibits key regulators of fatty acid metabolism. **(A, B)** GSEA of the expression proﬁle of HCCC9810 cells upon overexpression of KDM5C using a fatty acid metabolic process-associated signature **(A)** and a fatty acid biosynthetic process-associated signature **(B)**. **(C)** Summary of the functional categories of genes signiﬁcantly enriched in HCCC9810 cells transduced with KDM5C-expressing vector. Analyses were performed on the differentially expressed genes in HCCC9810 cells by KDM5C overexpression using DAVID. **(D-E)** Colony formation assays of HCCC9810 **(D)** and CCLP1 **(E)** cells upon knockdown of KDM5C. **(F-G)** Transwell assays were performed to detect the invasion ability of HCCC9810 **(F)** and CCLP1 **(G)** cells when KDM5C was knockdown. Scale bars, 100 μm. **(H)** Cellular free fatty acid was measured in HCCC9810 when KDM5C was knockdown. **p* < 0.05, ***p* < 0.01, ****p* < 0.001. All results are from three independent experiments.

**Figure S3**

**
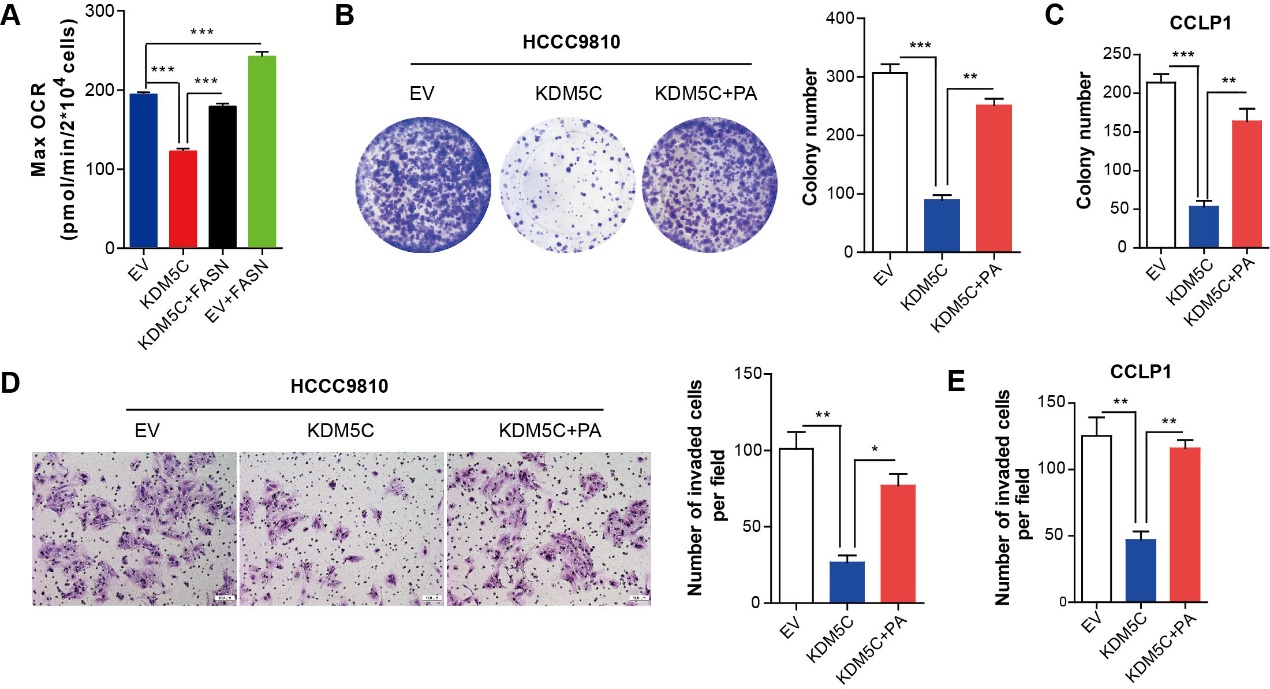
**

**Fig. S3** KDM5C inhibited ICC cell growth and invasion *via* modulating de novo fatty acid synthesis. **(A)** Oxygen consumption rates (OCR) was measured after recovering expression of FASN in control or KDM5C-overexpressed cells. **(B-C)** Colony formation assays were performed in HCCC9810 **(B)** and CCLP1 **(C)** cells with EV or KDM5C overexpression or KDM5C overexpression plus palmitate (200 μM) supplement. **(D-E)** Transwell assays were performed to detect the invasion ability of HCCC9810 **(D)** and CCLP1 **(E)** cells when EV or KDM5C was overexpressed or KDM5C was overexpressed plus palmitoleic acid (200 μM) supplement. Data are presented as the mean ± SD. **p* < 0.05, ***p* < 0.01, ****p* < 0.001. All results are from three independent experiments.

**Supplementary Tables**

| **Table S1 Univariate and Multivariate Analysis of Factors Associated with Survival and Recurrence** | | | | | | | | | |
| --- | --- | --- | --- | --- | --- | --- | --- | --- | --- |
| Features | Overall survival | | | |  | Time to recurrence | | | |
|  |  | Multivariate | | |  |  | Multivariate | | |
|  | Univariate,  *P* value | Hazard Ratio | 95%  CI | *P* value |  | Univariate,  *P* value | Hazard Ratio | 95%  CI | *P* value |
| Gender (male vs female) | 0.988 |  |  | NS |  | 0.417 |  |  | NS |
| Age (＞50 vs ≤50) | 0.421 |  |  | NS |  | 0.755 |  |  | NS |
| HBsAg (negative vs positive) | 0.380 |  |  | NS |  | 0.749 |  |  | NS |
| HBcAb (negative vs positive) | 0.138 |  |  | NS |  | 0.908 |  |  |  |
| Liver cirrhosis:yes vs. no | 0.422 |  |  | NS |  | 0.760 |  |  | NS |
| CA19-9 (＞37U/ml vs ≤37U/ml) | **0.012** | 1.581 | 1.039-2.406 | **0.032** |  | **0.003** | 1.899 | 1.174-3.073 | **0.009** |
| Preoperative ALT:≤75 vs. ＞75 U/L | 0.350 |  |  | NS |  | 0.549 |  |  | NS |
| Tumor size:≤5 vs.＞5 cm | 0.582 |  |  | NS |  | 0.365 |  |  | NS |
| Tumor number (solitary vs multiple) | 0.457 |  |  | NS |  | 0.421 |  |  |  |
| Tumor encapsulation: complete vs. none | 0.305 |  |  | NS |  | 0.387 |  |  | NS |
| Tumor thrombosis (yes vs no) | 0.088 |  |  | NS |  | 0.081 |  |  | NS |
| Regional lymph node metastasis(yes vs no) | **0.002** |  |  | NS |  | 0.449 |  |  |  |
| Tumor differentiation (III+IV vs I+II) | **0.007** | 1.613 | 1.061-2.452 | **0.025** |  | **0.003** | 1.878 | 1.168-3.021 | **0.009** |
| TNM stage (III+IVa vs I+II) | **＜0.001** | 1.746 | 1.027-2.970 | **0.040** |  | **0.002** | 1.832 | 1.047-3.206 | **0.031** |
| KDM5C (high vs low) | **0.002** |  |  | NS |  | **0.030** |  |  | NS |
| FASN (high vs low) | **0.004** | 1.664 | 1.089-2.541 | **0.019** |  | **0.012** | 1.773 | 1.097-2.867 | **0.019** |
| Combine KDM5C and FASN | **0.001** | 1.325 | 1.099-1.598 | **0.003** |  | **0.014** | 1.297 | 1.059-1.589 | **0.012** |

**Table S2. Comparison of clinicopathologic profiles between low and high KDM5C or FASN expression in ICC patients (n=190)**

| Variables | KDM5C (N=190) | | | | |  | FASN (N=190) | | | | |
| --- | --- | --- | --- | --- | --- | --- | --- | --- | --- | --- | --- |
|  | Low (N=95) | | High (N=95) | | P |  | Low (N=95) | | High (N=95) | | P |
|  | No. of patients | % | No. of patients | % |  |  | No. of patients | % | No. of patients | % |  |
| Gender^*^ |  |  |  |  |  |  |  |  |  |  |  |
| Female | 36 | 37.9% | 39 | 41.5% | 0.656 |  | 35 | 36.8% | 40 | 42.1% | 0.458 |
| Male | 59 | 62.1% | 56 | 58.5% |  |  | 60 | 63.2% | 55 | 57.9% |  |
| Age^*^ |  |  |  |  |  |  |  |  |  |  |  |
| ≥50 | 70 | 73.7% | 75 | 78.9% | 0.394 |  | 71 | 74.7% | 74 | 77.9% | 0.609 |
| ＜50 | 25 | 26.3% | 20 | 21.1% |  |  | 24 | 25.3% | 21 | 22.1% |  |
| HBsAg^*^ |  |  |  |  |  |  |  |  |  |  |  |
| Negative | 66 | 70.2% | 63 | 66.3% | 0.565 |  | 61 | 64.2% | 69 | 72.6% | 0.212 |
| Positive | 28 | 29.8% | 32 | 33.7% |  |  | 34 | 35.8% | 26 | 27.4% |  |
| Cirrhosis^*^ |  |  |  |  |  |  |  |  |  |  |  |
| No | 73 | 76.8% | 65 | 68.4% | 0.193 |  | 67 | 70.5% | 71 | 74.7% | 0.515 |
| Yes | 22 | 23.2% | 30 | 31.6% |  |  | 28 | 29.5% | 24 | 25.3% |  |
| ALT(U/L)^*^ |  |  |  |  |  |  |  |  |  |  |  |
| ≤ 75 | 75 | 78.9% | 74 | 77.9% | 0.860 |  | 74 | 77.9% | 75 | 78.9% | 0.860 |
| ＞75 | 20 | 21.1% | 21 | 22.1% |  |  | 21 | 22.1% | 20 | 21.1% |  |
| Tbil (μmol/dl) ^*^ | |  |  |  |  |  |  |  |  |  |  |
| ≤ 17.1 | 65 | 68.4% | 72 | 75.8% | 0.257 |  | 70 | 73.7% | 67 | 70.5% | 0.627 |
| ＞17.1 | 30 | 31.6% | 23 | 24.2% |  |  | 25 | 26.3% | 28 | 29.5% |  |
| GGT (U/L) | |  |  |  |  |  |  |  |  |  |  |
| ≤ 60 | 48 | 50.5% | 57 | 60.0% | 0.189 |  | 57 | 60.0% | 48 | 50.5% | 0.189 |
| ＞60 | 47 | 49.5% | 38 | 40.0% |  |  | 38 | 40.0% | 47 | 49.5% |  |
| CA19-9 (U/ml) ^*^ | |  |  |  |  |  |  |  |  |  |  |
| ≤ 37 | 41 | 43.2% | 49 | 51.6% | 0.245 |  | 44 | 46.3% | 46 | 48.4% | 0.771 |
| ＞37 | 54 | 56.8% | 46 | 48.4% |  |  | 51 | 53.7% | 49 | 51.6% |  |
| Tumor size (cm) ^*^ | |  |  |  |  |  |  |  |  |  |  |
| ≤5 | 42 | 44.2% | 47 | 49.5% | 0.467 |  | 49 | 51.6% | 40 | 42.1% | 0.191 |
| ＞5 | 53 | 55.8% | 48 | 50.5% |  |  | 46 | 48.4% | 55 | 57.9% |  |
| Tumor number^*^ | |  |  |  |  |  |  |  |  |  |  |
| Single | 86 | 90.5% | 90 | 94.7% | 0.267 |  | 86 | 90.5% | 90 | 94.7% | 0.267 |
| Multiple | 9 | 9.5% | 5 | 5.3% |  |  | 9 | 9.5% | 5 | 5.3% |  |
| Tumor encapsulation^*^ | |  |  |  |  |  |  |  |  |  |  |
| None | 81 | 85.3% | 87 | 91.6% | 0.174 |  | 83 | 87.4% | 85 | 89.5% | 0.650 |
| Complete | 14 | 14.7% | 8 | 8.4% |  |  | 12 | 12.6% | 10 | 10.5% |  |
| Tumor thrombus^*^ | |  |  |  |  |  |  |  |  |  |  |
| No | 87 | 91.6% | 79 | 83.2% | 0.081 |  | 83 | 87.4% | 83 | 87.4% | 1.000 |
| Yes | 8 | 8.4% | 16 | 16.8% |  |  | 12 | 12.6% | 12 | 12.6% |  |
| Tumor differentiation^*^ | |  |  |  |  |  |  |  |  |  |  |
| I+II | 57 | 60.0% | 69 | 72.6% | 0.065 |  | 62 | 65.3% | 64 | 67.4% | 0.759 |
| III+IV | 38 | 40.0% | 26 | 27.4% |  |  | 33 | 34.7% | 31 | 32.6% |  |
| pTNM stage^*^ | |  |  |  |  |  |  |  |  |  |  |
| I+II | 23 | 24.2% | 39 | 41.1% | **0.013** |  | 37 | 38.9% | 25 | 26.3% | 0.063 |
| III+IVa | 72 | 75.8% | 56 | 58.9% |  |  | 58 | 61.1% | 70 | 73.7% |  |
| Regional lymph node metastasis^*^ | | | |  |  |  |  |  |  |  |  |
| Negative | 74 | 77.9% | 87 | 91.6% | **0.009** |  | 83 | 87.4% | 78 | 82.1% | 0.313 |
| Positive | 21 | 22.1% | 8 | 8.4% |  |  | 12 | 12.6% | 17 | 17.9% |  |

p <0.05 was considered statistically significant.

* Chi-square test or Fisher exact.

Abbreviations: HBsAg, hepatitis B s-antigen; HBcAb, hepatitis B c-antibody; ALT, alanine aminotransferase; Tbil, total bilirubin, CA19-9, carbohydrate antigen 19-9; pTNM, pathological tumor, lymph node, metastasis classification.

**Supplementary Table S3. The list of PCR primer or shRNA targeting sequences used in this study**

| **Name** | **Sequence (5'-3')** | **Note** |
| --- | --- | --- |
| *KDM5C* | CCGAGGCTGTCAACTTTTGC | qRT-PCR |
| *KDM5C* | GTCAAAGGACTCAGCCCGAA | qRT-PCR |
| *β-Actin* | CATGTACGTTGCTATCCAGGC | qRT-PCR |
| *β-Actin* | CTCCTTAATGTCACGCACGAT | qRT-PCR |
| *shKDM5C#1* | TCGCAGAGAAATCGGGCATTT | shRNA |
| *shKDM5C#2* | AGTACCTGCGGTATCGGTATA | shRNA |
